# Quality of Life Before and at 6 and 12 Months After Permanent Cardiac Pacemaker Implantation and Recipients' Perspectives and Behaviors: A Cross-Sectional Study

**DOI:** 10.7759/cureus.105677

**Published:** 2026-03-23

**Authors:** Aikaterini Tsoni, Maria Polikandrioti, Antonia Kalogianni, Victoria Alikari, Georgios Vasilopoulos, Dimos Mastrogiannis, Angeliki Stamou, Theodoros Kapadohos

**Affiliations:** 1 Department of Nursing, University of West Attica, Athens, GRC

**Keywords:** adherence, anxiety, pacemaker, perspectives, quality of life

## Abstract

Introduction

Implantation of a permanent cardiac pacemaker (PPM) is critical for managing rhythm disorders and prolonging survival. Beyond clinical outcomes, implantation can affect patients’ quality of life (QoL), encompassing physical and mental dimensions. The present study aimed to evaluate QoL among patients undergoing PPM implantation, both before implantation and at 6 and 12 months post-implantation, as well as the associated perspectives and behaviors after 6 and 12 months.

Methods

In the present study, 112 patients undergoing PPM implantation were enrolled at a public hospital in Athens between 2023 and 2025. Participants were selected by the method of convenience sampling. Data collection was performed using the 36-Item Short Form Health Survey (SF-36). Scores for the Physical Component Summary (PCS) and the Mental Component Summary (MCS) were calculated using QoL scores that range from 0 to 100, with higher scores indicating higher QoL. Statistical analysis was performed using IBM SPSS Statistics for Windows, Version 28 (Released 2021; IBM Corp., Armonk, NY, USA). Statistical significance was set at p < 0.05.

Results

The PCS presented a mean of 36.2 ± 4.6, and the MCS a mean of 40.9 ± 13.5 before implantation. Post-implantation at 6 and 12 months, the mean PCS score was 34.7 ± 4.9 and 35.3 ± 4.3, respectively, while the mean MCS score was 55.6 ± 8.3 and 59.3 ± 7.7, respectively. Better PCS at 6 and 12 months was associated with adherence to regular follow-up (p = 0.013 and p = 0.027), antiarrhythmic therapy (p = 0.004 and p = 0.041), assistance at home (p = 0.026 and p = 0.012), and being physically active (p = 0.022 and p = 0.004). Better MCS at 6 and 12 months was associated with anxiety about rhythm disorders (p = 0.013 and p = 0.016), participation in social activities (p = 0.001 and p = 0.001), assistance at home (p = 0.001 and p = 0.019), and being physically active (p = 0.001 and p = 0.001).

Conclusion

Assessing both QoL and patients’ perspectives before and after PPM implantation provides valuable insights into the real-world impact of this therapy and informs patient-centered care.

## Introduction

Permanent cardiac pacemakers (PPMs) are medical devices designed to maintain a normal cardiac rhythm. Globally, approximately 3 million individuals live with a PPM, and around 600,000 new implants are performed annually [1,2]. PPM implantation rates vary significantly by age and geographic region. In the United States, implantation rates are approximately 62 per 100,000 population [3]. In contrast, low- and middle-income countries report substantially lower implantation rates, often fewer than 1 per 100,000 population [4]. Over the past 25 years, technological advancements and expanded clinical indications have established cardiac pacing as an active area of ongoing research. Since 1999, the Journal Europace has contributed significantly to this field by publishing over 1,300 articles addressing various aspects of cardiac pacing [5]. 

PPM implantation is a well-established cardiac intervention that warrants long-term follow-up to provide insights into device longevity and patient outcomes [1]. Assessing quality of life (QoL) offers valuable insight into the extent to which a patient’s clinical status affects both subjective experiences and objectively measurable symptoms. It also facilitates ongoing monitoring of disease progression and related changes over time. Pre-implant QoL assessments establish a baseline by capturing patients’ symptoms, functional status, mobility, comfort, independence, and overall well-being. This baseline serves as a reference point for meaningful comparison with post-procedure outcomes and post-implant QoL. From a research and quality-improvement perspective, pre- and post-implant QoL data contribute to evidence-based practice and guide the development of effective interventions that address the multidimensional needs of PPM recipients [6-8].

Traditionally, healthcare quality assessments have relied predominantly on medical perspectives; however, contemporary evaluation frameworks place greater emphasis on patient input, thereby broadening the focus beyond health outcomes alone. More specifically, assessments based on patient perspectives play a critical role in healthcare by influencing how individuals utilize medical services, engage with treatment options, and cope with their disease. Patient perspectives are increasingly integrated into research, providing evidence that can inform health policy, particularly in cases where patients’ views do not align with clinical priorities [8-10]. 

In cardiac pacing, clinical assessments focus on physiological measures and treatment efficacy, often overlooking the emotional and psychological dimensions of health [11]. Within the context of QoL, clinical measures such as heart rhythm stabilization and device functionality provide objective indicators of pacing effectiveness. Incorporating patients’ perspectives facilitates a comprehensive evaluation of outcomes, thereby supporting holistic care and informed clinical decision-making.

The aim of the present study was to evaluate QoL among patients undergoing PPM implantation, both before implantation and at 6 and 12 months post-implantation, as well as to assess patients’ perspectives and behaviors at 6 and 12 months post-implantation.

## Materials and methods

Design, setting, and period of the study

**Figure 1:**
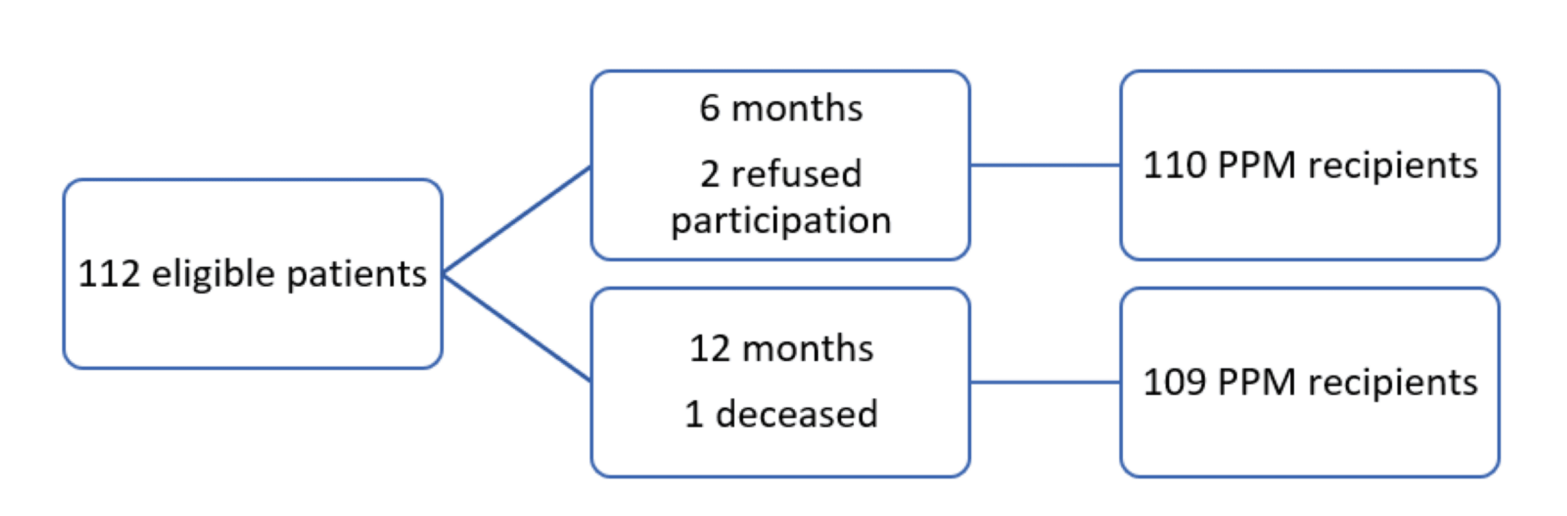
Flow diagram of patient selection PPM: Permanent Cardiac Pacemaker

In this longitudinal study, 112 patients undergoing PPM implantation were enrolled at a public hospital in Athens from 2023 to 2025. Participants were selected using convenience sampling. The initial study population comprised 112 patients; however, at the 6-month follow-up, two patients declined to participate further, reducing the sample size to 110. At the 12-month follow-up, one patient had died, resulting in a final sample of 109 participants (Figure 1).

Inclusion and exclusion criteria of the sample

The inclusion criteria were the ability to write and speak Greek, and to read and sign the informed consent form. The exclusion criteria were insertion of other devices, such as a defibrillator, and being diagnosed either with mental illness, receiving medication, or with cognitive disorders and sight or hearing impairment. Information regarding psychiatric comorbidity and cognitive disorders was obtained from patients’ medical records and cardiologist assessments. Finally, in the present study, only patients with traditional PPMs (conventional devices with leads inserted into the heart via veins) were enrolled, and not those with leadless PPMs (devices implanted directly into the heart without leads). In this study, participants with the following types of PPMs were included: (i) Ventricular Inhibited (VVI): A pacemaker mode that senses and paces the ventricle only, and inhibits output when intrinsic ventricular activity is detected. (ii) Dual-Chamber (DDD): Dual-chamber pacing and sensing with tracking of intrinsic atrial activity; the pacing rate is fixed unless changed manually. (iii) Dual-Chamber Rate-Modulated mode (DDDR): Same functions as DDD, plus rate-responsiveness, meaning it automatically increases the pacing rate during physical activity.

Data collection and procedure

Data were collected using the method of interviews at three time points: (a) before implantation (baseline); (b) 6 months after the implantation; and (c) 12 months after the implantation. More in detail, at baseline, QoL was measured prior to implantation upon hospital admission, while at 6 and 12 months, assessments were conducted when participants were visiting the hospital's outpatient clinics for scheduled follow-up. All measurements were conducted in a private office room at the hospital to guarantee confidentiality. The average time required to complete the questionnaire was approximately 20-30 minutes.

Data were collected using the 36-Item Short Form Health Survey (SF-36) and included patients’ perceptions and behaviors, as well as demographic and clinical characteristics, such as age, gender, type of PPM, and diagnosis before implantation. In terms of patients’ perceptions and behaviors, the following were included: adherence to scheduled follow-up visits; adherence to prescribed antiarrhythmic medication; anxiety related to heart rhythm disorders and device function; belief that life depends on PPM; belief that life depends on regular re-evaluations; maintenance of physical activity after PPM; and participation in social activities, receiving assistance at home, and carrying a special identification card (ID) listing pacemaker details.

QoL measurement

To assess QoL, the SF-36 Health Survey was used. The SF-36, introduced by Ware et al. [12] in 1993, assesses both physical and mental health and includes 36 items across eight domains: physical functioning; role limitations due to physical health; role limitations due to emotional problems; bodily pain; vitality; social functioning; mental health; and general health perceptions. Respondents answer the items using Likert-type scales. 

In addition, summary scores for the Physical Component Summary (PCS) and the Mental Component Summary (MCS) were calculated using the standard SF-36 (v 1.0). This procedure includes recoding items into the eight subscale scores, converting each subscale to a z-score using population norms, applying the published factor-score coefficients for the physical and mental components, and transforming the resulting component scores to T-scores, with a mean of 50 and a standard deviation of 10. Scores range from 0 to 100, with increasing values representing better-perceived QoL [12,13]. In the present study, the Greek version of the scale was used [13]. The use of the scale does not require permission (https://www.rand.org/health/surveys/mos/36-item-short-form/terms.html). 

Ethical considerations

This study received approval (protocol number 4843/13.03.23) from the Research Committee of the public hospital “General Hospital of Attica Sismanoglio-Amalia Fleming”, where it was conducted. Participants were informed by the researchers about the purposes of the study, the anonymity of the data, and that the data would be used only for the study. Written informed consent was obtained. All subjects were informed of their rights to refuse or discontinue participation in the study, according to the ethical standards of the Declaration of Helsinki (1989) of the World Medical Association.

Statistical analysis

Categorical variables are presented as absolute and relative (%) frequencies, whereas quantitative variables are presented as mean, standard deviation (±SD), median, and interquartile range (IQR). Independent samples t-test and analysis of variance (ANOVA), or Kruskal-Wallis and Mann-Whitney tests, were used to examine associations between QoL scores and patient characteristics, depending on whether normality assumptions were met. Normality was assessed using the Shapiro-Wilk test and visually through histograms. To detect differences or trends in patients’ QoL over time, repeated measures ANOVA models were applied. Mauchly's test of sphericity was assessed, and if the assumption did not hold, a correction with Greenhouse-Geisser's ε was considered. Cohen’s d effect sizes were also calculated. Effect sizes were classified based on Cohen’s criteria, with values of 0.2, 0.5, and 0.8 corresponding to small, medium, and large effects, respectively. A significance level of 5% was considered statistically significant. All statistical analyses were performed using IBM SPSS Statistics for Windows, Version 28 (Released 2021; IBM Corp., Armonk, NY, USA).

## Results

The majority of participants were male, representing 61 (54.5%) of the sample, while women were 51 (45.5%). In terms of age, 13 (11.6%) were 51-60 years old, 26 (23.2%) were 61-70, 35 (31.3%) were 71-80, and those over 80 years old were 38 (33.9%). In terms of pacemaker type, 48 (42.9%) had DDDR, and 38 (33.9%) had a diagnosis of atrial fibrillation before PPM insertion (Table 1).

**Table 1:** Demographic and clinical characteristics of the sample (N = 112 at baseline) VVI: Ventricular Inhibited; DDD: Dual-Chamber; DDDR: Dual-Chamber Rate-Modulated Mode; PPM: Permanent Cardiac Pacemaker

Characteristics	n (%)
Gender	
Male	61 (54.5%)
Female	51 (45.5%)
Age	
51-60	13 (11.6%)
61-70	26 (23.2%)
71-80	35 (31.3%)
80	38 (33.9%)
Type of Pacemaker	
VVI	37 (33.0%)
DDDR	48 (42.9%)
DDD	27 (24.1%)
Diagnosis Leading to PPM Implantation	
Atrial fibrillation	38 (33.9%)
Mobitz type II atrioventricular block	20 (17.9%)
Sick sinus syndrome	29 (25.9%)
Complete atrioventricular block	25 (22.3%)

Description of perspectives and behaviors at 6- and 12-month post-implantation

Table 2 presents the results regarding patients’ perspectives and behaviors, declared at follow-up at 6 and 12 months after PPM implantation.

**Table 2:** Description of responses at 6- and 12-month post-implantation PPM: Permanent Cardiac Pacemaker; ID Card: Identification Card

	6 months (n =110), n(%)	12 months (n = 109), n(%)
Adherence to scheduled follow-up visits		
Very	49 (44.5%)	51 (46.8%)
Fairly	56 (50.9%)	55 (50.5%)
Slightly	5 (4.5%)	3 (2.8%)
Not at all	0 (0.0%)	0 (0.0%)
Adherence to prescribed antiarrhythmic medication		
Very	56 (50.9%)	55 (50.5%)
Fairly	51 (46.4%)	51 (46.8%)
Slightly	3 (2.7%)	3 (2.8%)
Not at all	0 (0.0%)	0 (0.0%)
Anxiety about heart rhythm disorders		
Very	18 (16.4%)	5 (4.6%)
Fairly	43 (39.1%)	29 (26.6%)
Slightly	45 (40.9%)	73 (67.0%)
Not at all	4 (3.6%)	2 (1.8%)
Anxiety about device function		
Very	27 (24.5%)	5 (4.6%)
Fairly	58 (52.7%)	49 (45.0%)
Slightly	23 (20.9%)	54 (49.5%)
Not at all	2 (1.8%)	1 (0.9%)
Belief that life depends on PPM		
Very	16 (14.5%)	8 (7.3%)
Fairly	79 (71.8%)	55 (50.5%)
Slightly	14 (12.7%)	45 (41.3%)
Not at all	1 (0.9%)	1 (0.9%)
Belief that life depends on regular re-evaluations		
Very	15 (13.6%)	7 (6.4%)
Fairly	67 (60.9%)	42 (38.5%)
Slightly	27 (24.5%)	59 (54.1%)
Not at all	1 (0.9%)	1 (0.9%)
Remain physically active after PPM		
Very	21 (19.1%)	31 (28.4%)
Fairly	59 (53.6%)	57 (52.3%)
Slightly	27 (24.5%)	20 (18.3%)
Not at all	3 (2.7%)	1 (0.9%)
Participation in social activities		
Very	31 (28.2%)	45 (41.3%)
Fairly	51 (46.4%)	47 (43.1%)
Slightly	26 (23.6%)	16 (14.7%)
Not at all	2 (1.8%)	1 (0.9%)
Assisted at home (Yes)	84 (76.4%)	82 (75.2%)
Carrying an ID card		
Yes	32 (29.1%)	34 (31.2%)
No	37 (33.6%)	24 (22.0%)
Sometimes	41 (37.3%)	51 (46.8%)

With respect to adherence to scheduled follow-up visits, results indicate a high degree of compliance at both time points. At 6 months, 44.5% of patients reported attending follow-up appointments “very” regularly, while 50.9% reported “fairly” regular attendance. At 12 months, these proportions remained almost unchanged, with 46.8% responding “very” and 50.5% “fairly.” Similarly, high levels of adherence to prescribed antiarrhythmic medication were observed. At 6 months, 50.9% reported taking their medication “very” consistently, and 46.4% “fairly” consistently; these percentages remained largely unchanged at 12 months.

Self-reported anxiety related to heart rhythm disorders at 6 months was rated as “very” by 16.4% and “fairly” by 39.1%. At 12 months, the corresponding proportions decreased to 4.6% and 26.6%, respectively. A similar trend was observed for anxiety concerning device function: at 6 months, 24.5% reported “very” high anxiety and 52.7% “fairly” high anxiety; by 12 months, these proportions had decreased to 4.6% and 45.0%, respectively, accompanied by a substantial increase in the proportion of participants reporting being “slightly” anxious (49.5%).

An interesting finding relates to perceived dependence on the device and on regular re-evaluations (follow-up). At 6 months, 14.5% felt their life depended “very” much on the pacemaker, and 71.8% “fairly” much. At 12 months, these proportions decreased to 7.3% and 50.5%, respectively, accompanied by a notable increase in those perceiving only “slight” dependence (41.3%). A similar pattern was observed regarding dependence on regular follow-ups (re-evaluations): from 13.6% “very” and 60.9% “fairly” at 6 months, the proportions decreased to 6.4% and 38.5% at 12 months, while the percentage reporting “slight” dependence increased to 54.1%.

Regarding physical activity after PPM implantation, the proportion of patients describing themselves as “very” physically active rose from 19.1% at 6 months to 28.4% at 12 months. Participants’ social activity appeared to improve over time. At 6 months, 28.2% reported participating “very” actively in social activities, and 46.4% “fairly.” By 12 months, these proportions rose to 41.3% and 43.1%, respectively, while the percentages of those reporting “slight” or “no” participation declined.

In parallel, approximately three-quarters of participants reported having someone at home to assist with daily activities at both time points (76.4% at 6 months and 75.2% at 12 months). Concerning possession of a special ID card listing pacemaker details at 6 months, 29.1% reported carrying the card, 33.6% not to carry, and 37.3% carrying it “sometimes.” At 12 months, there was a slight increase in those who reported “always” carrying the card (31.2%), accompanied by a decrease in those who reported “never” carrying it.

QoL measurement

Regarding baseline summary QoL scores, the PCS presented a mean score of 36.2 and a standard deviation of 4.6, and a median of 36.6, showing a clear impairment in physical health. The MCS showed a mean of 40.9 and a standard deviation of 13.5, and a median of 41.5, which, although also below population norms, was comparatively higher than the physical health component (Table 3).

**Table 3:** Description of patients’ QoL over time Calculated using repeated measures ANOVA to assess time trends; significance level p < 0.05. SD: Standard Deviation; IQR: Interquartile Range; QoL: Quality of Life; ANOVA: Analysis of Variance

	Baseline (n = 112)		6 m (n = 110)		12 m (n = 109)		-		
SF-36 (Range: 0-100)	Mean (SD)	Median (IQR)	Mean (SD)	Median (IQR)	Mean (SD)	Median (IQR)	F-statistic	p-value	Cohen’s d
Physical Component Summary (PCS)	36.2 (4.6)	36.6 (33.5-38.8)	34.7 (4.9)	34.7 (32.1-38.3)	35.3 (4.3)	35.5 (32.4-38.9)	6.915	<0.001	-0.20
Mental Component Summary (MCS)	40.9 (13.5)	41.5 (29.3-50.9)	55.6 (8.3)	56.7 (51.5-61.1)	59.3 (7.7)	59.5 (55.5-65.2)	225.7	<0.001	1.67

Longitudinal changes in QoL (trends)

The PCS exhibited minor fluctuations, with a slight decline at 6 months (36.2 → 34.7), followed by a small increase at 12 months (35.3), suggesting relative stability in physical health. In contrast, the MCS showed a substantial and continuous improvement, rising from 40.9 at baseline to 55.6 at 6 months and 59.3 at 12 months. Likewise, Cohen’s d effect sizes indicated a small decrease in PCS over time (d = -0.20), whereas MCS demonstrated a very large improvement (d = 1.67).

Patients' perspectives and behaviors associated with QoL at 6 and 12 months after PPM implantation

On the PCS scale, multiple statistically significant associations were recorded at both 6 and 12 months post-implantation. Adherence to regular follow-up visits was positively associated with better PCS scores (6 months: p = 0.013, M = 35.7 for “very” vs. 33.6 for “fairly”; 12 months: p = 0.027, M = 36.3 vs. 34.4). Similarly, adherence to prescribed antiarrhythmic therapy demonstrated a statistically significant association (6 months: p = 0.004, M = 35.8 vs. 33.4; 12 months: p = 0.041, M = 36.1 vs. 34.3).

The presence of a household member to assist with daily activities was linked to lower PCS scores compared to those without such help (6 months: p = 0.026, M = 34.1 vs. 36.5; 12 months: p = 0.012, M = 34.8 vs. 37.1). At 12 months, lower anxiety regarding device function was positively associated with higher PCS (p = 0.007, M = 36.5 for “slightly-not at all” anxiety vs. 34.1-34.7 in the other categories).

Greater participation in social obligations was related to better PCS (p = 0.001, M = 36.7 for “very” participation, 35.3 for “fairly,” and only 31.8 for “slightly-not at all”). Physical activity emerged as a strong factor (6 months: p = 0.022, M = 36.1 for “very” vs. 32.4 for “slightly-not at all”; 12 months: p = 0.004, M = 37.4 vs. 33.0). Finally, possession and carrying of a pacemaker ID was positively associated with PCS at 6 months (p = 0.017, M = 35.8 vs. 32.6) (Table 4).

**Table 4:** Patients' perspectives and behaviors associated with QoL at 6 and 12 months after implantation * Statistically significantly different (p < 0.05) from the other categories after Bonferroni correction for multiple comparisons; ^1 ^p-value calculated from t-test; ^2 ^p-value calculated from ANOVA. ANOVA: Analysis of Variance; SD: Standard Deviation; IQR: Interquartile Range; PCS: Physical Component Summary; PPM: Permanent Cardiac Pacemaker; QoL: Quality of Life; ID Card: Identification Card

	Physical Component Summary (PCS)							
	6 months				12 months			
	Mean (SD)	Median (IQR)	F/t statistic	p-value	Mean (SD)	Median (IQR)	F/t statistic	p-value
Adherence to scheduled follow-up visits	-		2.152	0.013^1*^	-		5.441	0.027^1*^
Very	35.7 (5.0)	36.8 (33.0-39.0)	-	-	36.3 (4.1)	36.8 (32.9-39.1)	-	-
Fairly	33.6 (4.7)	33.3 (31.1-36.5)	-	-	34.4 (4.2)	34.0 (32.3-37.2)	-	-
Adherence to prescribed antiarrhythmic medication	-		2.465	0.004^1*^	-		4.846	0.041^1*^
Very	35.8 (5.0)	36.8 (33.0-39.1)	-	-	36.1 (4.0)	36.4 (33.7-39.1)	-	-
Fairly	33.4 (4.6)	32.7 (31.1-36.5)	-	-	34.3 (4.4)	34.0 (32.2-37.7)	-	-
Anxiety about rhythm disorder	-		0.426	0.533^2^	-		1.159	0.180^2^
Very	33.7 (4.3)	34.2 (30.5-37.5)	-	-	33.5 (1.7)	33.9 (32.3-34.5)	-	-
Fairly	35.1 (3.6)	35.0 (32.4-37.7)	-	-	34.6 (3.7)	34.0 (32.5-36.5)	-	-
Slightly/Not at all	34.7 (5.9)	35.1 (31.2-39.0)	-	-	35.7 (4.6)	36.7 (32.6-39.1)	-	-
Anxiety about device function	-		0.494	0.330^2^	-		4.215	0.007^2*^
Very	34.0 (4.2)	34.7 (31.2-37.5)	-	-	34.7 (3.3)	34.5 (32.3-35.7)	-	-
Fairly	34.7 (4.5)	34.1 (32.2-38.3)	-	-	34.1 (3.9)	33.9 (32.1-36.5)	-	-
Slightly/Not at all	35.4 (6.4)	36.7 (31.6-40.5)	-	-	36.5 (4.5)	38.1 (33.8-39.4)*	-	-
Belief that life depends on PPM	-		2.774	0.083^2^	-		0.443	0.562^2^
Very	34.6 (4.5)	35.0 (31.5-37.7)	-	-	33.9 (4.4)	34.3 (31.1-37.5)	-	-
Fairly	34.2 (4.9)	34.2 (31.9-37.7)	-	-	35.4 (3.5)	35.1 (32.5-38.9)	-	-
Slightly/Not at all	37.4 (4.3)	38.8 (33.2-40.6)	-	-	35.5 (5.2)	36.3 (32.6-39.1)	-	-
Belief that life depends on regular re-evaluations	-		0.763	0.429^2^	-		0.059	0.746^2^
Very	34.6 (4.6)	35.3 (30.5-37.9)	-	-	35.1 (3.4)	34.5 (31.3-38.5)	-	-
Fairly	34.3 (4.7)	34.2 (32.1-37.7)	-	-	35.2 (3.5)	34.7 (32.5-38.1)	-	-
Slightly/Not at all	35.6 (5.3)	36.4 (31.9-40.4)	-	-	35.3 (4.8)	35.9 (32.4-39.1)	-	-
Belief that remaining physically active after PPM	-		4.492	0.022^2*^	-		7.594	0.004^2*^
Very	36.1 (4.0)	36.8 (35.2-39.0)	-	-	37.4 (3.0)	38.1 (35.7-39.1)*	-	-
Fairly	35.2 (4.7)	34.2 (32.1-39.0)	-	-	35.1 (4.4)	34.3 (32.4-39.3)	-	-
Slightly/Not at all	32.4 (5.3)	32.6 (29.2-36.6)	-	-	33.0 (4.6)	33.5 (31.3-36.4)	-	-
Participation in social activities	-		2.433	0.054^2^	-		9.508	0.001^2*^
Very	35.9 (4.1)	36.7 (32.2-39.1)	-	-	36.7 (3.4)	37.6 (34.2-38.9)	-	-
Fairly	35.0 (4.8)	34.2 (32.4-38.8)	-	-	35.3 (4.4)	35.1 (32.4-39.5)	-	-
Slightly/Not at all	32.6 (5.3)	33.2 (30.3-37.5)	-	-	31.8 (4.5)	32.0 (29.8-34.0)	-	-
Assisted at home	-		5.07	0.026^1*^	-		6.067	0.012^1*^
Yes	34.1 (5.1)	33.7 (31.2-37.7)	-	-	34.8 (4.4)	34.3 (32.3-38.5)	-	-
No	36.5 (3.8)	37.1 (33.9-39.1)	-	-	37.1 (3.6)	38.2 (35.5-39.3)	-	-
Carrying an ID card	-		4.696	0.017^2*^	-		2.126	0.201^2^
Yes	35.8 (4.0)	36.7 (33.0-39.0)	-	-	36.3 (3.6)	36.5 (33.7-39.1)	-	-
No	32.6 (5.4)	32.5 (30.9-36.1)	-	-	34.0 (5.2)	33.7 (32.0-37.2)	-	-
Sometimes	35.5 (4.6)	35.7 (32.5-38.9)	-	-	35.3 (4.2)	35.7 (32.4-38.9)	-	-

In the MCS scale, several statistically significant associations were identified at both 6- and 12-month post-implantation. Higher anxiety about rhythm disorders was associated with lower MCS scores, both at 6 months (p = 0.013, M = 50.0 for “very” anxiety vs. 57.4 for “slightly/not at all”) and at 12 months (p = 0.016, M = 57.4 vs. 60.6). The absence of a household member to assist with daily activities was linked to higher MCS (6 months: p = 0.001, M = 59.8 vs. 54.1; 12 months: p = 0.019, M = 61.7 vs. 58.6). The belief in remaining physically active after implantation also emerged as an important factor, with highly active patients achieving the highest MCS scores (6 months: p = 0.001, M = 60.6 for “very active” vs. 46.7 for “slightly/not at all”; 12 months: p = 0.001, M = 63.7 vs. 51.9). Participation in social activities showed a strong positive association at both time points (6 months: p = 0.001, M = 61.1 for “very,” 57.1 for “fairly,” and 46.7 for “slightly/not at all”; 12 months: p = 0.001, M = 62.7, 59.2, and 50.7, respectively) (Table 5).

**Table 5:** Patients' perspectives and behaviors associated with QoL at 6 and 12 months after implantation * Statistically significantly different (p < 0.05) from the other categories after Bonferroni correction for multiple comparisons; ^1^ p-value calculated from t-test; ^2^ p-value calculated from ANOVA. ANOVA: Analysis of Variance; SD: Standard Deviation; IQR: Interquartile Range; PCS: Physical Component Summary; PPM: Permanent Cardiac Pacemaker; QoL: Quality of Life; ID Card: Identification Card

	Mental Component Summary (MCS)							
	6 months				12 months			
	Mean (SD)	Median (IQR)	F/t statistic	p-value	Mean (SD)	Median (IQR)	F/t statistic	p-value
Adherence to scheduled follow-up visits	-		1.42	0.168^1^	-		2.356	0.215^1^
Very	57.0 (6.5)	57.9 (54.0-61.1)	-	-	60.9 (5.4)	60.7 (57.2-65.9)	-	-
Fairly	54.7 (9.2)	55.6 (49.8-60.4)	-	-	58.7 (8.5)	59.1 (55.0-64.5)	-	-
Adherence to antiarrhythmic medication	-		1.255	0.290^2^	-		1.022	0.584^1^
Very	56.8 (6.4)	57.7 (53.9-61.1)	-	-	60.5 (5.6)	60.5 (56.4-65.4)	-	-
Fairly	54.8 (9.5)	55.7 (50.2-61.7)	-	-	59.0 (8.6)	59.1 (55.6-65.2)	-	-
Anxiety about rhythm disorders	-		5.513	0.013^2*^	-		5.873	0.016^2*^
Very	50.0 (11.8)	48.8 (43.5-59.7)	-	-	57.4 (8.6)	54.8 (52.8-56.4)	-	-
Fairly	55.6 (6.0)	55.5 (52.6-59.3)	-	-	56.5 (8.6)	57.5 (54.9-60.8)	-	-
Slightly/Not at all	57.4 (7.7)	58.1 (55.2-62.0)	-	-	60.6 (7.1)	60.8 (57.1-65.8)	-	-
Anxiety about device function	-		0.450	0.246^2^	-		0.337	0.766^2^
Very	52.6 (11.0)	52.9 (45.2-61.1)	-	-	60.1 (8.1)	58.0 (56.4-62.7)	-	-
Fairly	56.2 (7.6)	55.8 (52.6-61.4)	-	-	58.6 (8.7)	59.0 (54.9-64.9)	-	-
Slightly/Not at all	57.4 (5.1)	57.3 (55.1-61.1)	-	-	59.9 (6.7)	60.4 (56.4-65.3)	-	-
Belief that life depends on PPM	-		4.877	0.058^2^	-		0.889	0.215^2^
Very	51.3 (9.9)	49.0 (44.4-58.4)	-	-	56.3 (9.6)	53.6 (48.7-63.9)	-	-
Fairly	56.0 (7.7)	57.1 (52.9-61.0)	-	-	59.1 (6.9)	58.8 (55.6-62.7)	-	-
Slightly/Not at all	57.9 (8.2)	60.0 (52.6-63.7)	-	-	60.2 (8.2)	62.2 (55.8-66.0)	-	-
Belief that life depends on regular re-evaluations	-		2.137	0.074^2^	-		1.366	0.091^2^
Very	51.5 (10.3)	49.1 (43.5-59.7)	-	-	57.8 (9.3)	56.4 (49.1-67.0)	-	-
Fairly	55.9 (7.7)	56.2 (52.9-60.8)	-	-	58.0 (7.7)	57.5 (55.1-60.9)	-	-
Slightly/Not at all	56.9 (8.1)	58.6 (52.2-62.7)	-	-	60.9 (6.8)	61.9 (57.7-65.8)	-	-
Belief that remaining physically active after PPM	-		38.815	0.001^2*^	-		20.044	0.001^2*^
Very	60.6 (4.6)	61.1 (59.3-63.8)	-	-	63.7 (6.9)	65.8 (62.7-67.0)	-	-
Fairly	58.1 (5.8)	58.3 (54.6-61.3)	-	-	59.7 (5.1)	59.0 (55.6-61.9)	-	-
Slightly/Not at all	46.7 (8.1)	49.0 (40.9-53.5)	-	-	51.9 (9.2)	54.7 (49.1-57.1)	-	-
Participation in social activities	-		41.123	0.001^2*^	-		20.417	0.001^2*^
Very	61.1 (4.5)	61.3 (59.3-63.8)	-	-	62.7 (6.6)	64.4 (59.8-66.6)	-	-
Fairly	57.1 (5.8)	57.3 (53.0-60.0)	-	-	59.2 (4.9)	58.7 (55.5-61.3)	-	-
Slightly/Not at all	46.7 (8.2)	49.1 (39.4-54.3)	-	-	50.7 (9.8)	54.0 (48.3-56.4)	-	-
Assisted at home	-		10.031	0.001^1*^	-		5.496	0.019^1*^
Yes	54.1 (8.8)	55.0 (49.3-59.6)	-	-	58.6 (7.7)	58.7 (55.0-63.9)	-	-
No	59.8 (4.1)	60.3 (57.1-62.0)	-	-	61.7 (7.3)	63.3 (58.8-66.0)	-	-
Carrying an ID card	-		0.257	0.827^2^	-		0.297	0.973^2^
Yes	56.5 (7.9)	57.1 (51.6-61.1)	-	-	60.1 (6.3)	58.7 (56.4-64.9)	-	-
No	55.2 (9.6)	56.5 (50.2-61.5)	-	-	57.8 (11.1)	59.4 (55.1-65.0)	-	-
Sometimes	55.2 (7.5)	56.1 (51.0-60.0)	-	-	59.5 (6.5)	60.2 (55.4-65.2)	-	-

## Discussion

According to the results, the PCS exhibited a slight decline at 6 months, followed by a modest increase at 12 months, suggesting relative stability in physical health. In contrast, the MCS showed substantial and continuous improvement at both 6 and 12 months. In other words, while physical functioning remained relatively stable, the mental profile of patients improved markedly within the first year following implantation. The limited improvement observed in physical QoL post-implantation is attributable to several interrelated factors. Though pacemakers effectively correct bradyarrhythmia, they do not reverse the underlying structural or functional cardiac abnormalities. Moreover, device therapy does not necessarily enhance exercise tolerance or muscular strength. Furthermore, many PPM recipients are elderly and have multiple comorbidities, which may limit measurable gains in physical functioning. Notably, in the present study, 65.2% were over 70 years old. Finally, postoperative physical recovery may be slow, as patients often experience fear of living with the device and self-impose physical limitations [7,8,11,14,15].

The study of van Nuland et al. showed that patients who underwent PPM implantation prior to transcatheter aortic valve implantation exhibited lower physical scores over time compared to those who did not [16]. Likewise, a relevant study in the Greek PPM population (n = 150) demonstrated higher QoL scores in emotional well-being and the lowest ones in physical functioning, as measured by SF-36 [8]. Also, a recent study in Greek PPM recipients highlighted that psychological factors, such as anxiety and depression, influenced QoL, underscoring the importance of addressing mental health alongside physical health in this population [7]. In China, Lin et al. showed a 39.92% prevalence of depression among PPM recipients (n = 206) during the COVID-19 pandemic [17]. Fatigue had the strongest negative association with QoL. Li et al. indicated that PPM implantation is associated with significant improvements in patients’ QoL (n = 84) [18]; however, in patients with permanent atrial fibrillation and ventricular arrest, device insertion did not reduce the incidence of sudden cardiac death, cardiovascular events, or stroke. Approximately five years ago, Adoubi et al., in a sample of 97 PPM recipients, demonstrated symptom improvement, as reflected by a favorable AQUAREL score, whereas the SF-12 revealed only modest outcomes, particularly in the physical component (41.3 ± 9.1) compared to the mental component (79 ± 33.3) [19]. Willy et al., in a systematic search and meta-analysis examining cardiac device implantation, concluded that current evidence remains limited, despite available data indicating potential beneficial effects [20]. The authors also underscored the importance of routinely assessing QoL in clinical trials to inform implantation decisions, patient education, and shared decision-making [20].

Adherence to regular follow-up visits and to antiarrhythmic therapy was positively associated with better QoL in the physical component at both 6 and 12 months. This finding suggests that patients who maintained treatment adherence and engaged in ongoing clinical monitoring experienced better physical health outcomes over time. Interestingly, treatment adherence contributes to enhanced symptom control, fewer arrhythmic episodes, and greater functional capacity, thereby optimizing long-term physical well-being [7,8,21]. In a Greek sample of PPM recipients (n = 150), attendance at scheduled follow-up visits was associated with better QoL in physical and emotional roles, in social functioning, and general health [8]. Follow-up visits for PPM recipients enable individualized care, effective communication, and comprehensive counseling, allowing patients to articulate their concerns, while healthcare providers can align treatment with patient values [7,8]. In addition, follow-ups generate essential clinical data (heart rate patterns, arrhythmia episodes, device function, and complications) that contribute to improved long-term management [8,22].

A finding directly related to the field of rhythm management was that patients who carried the PPM ID card reported better physical QoL at 6 months. This group of recipients may exhibit higher levels of engagement in health management. Additionally, possession of the card provides a sense of security and reassurance, facilitating greater confidence in performing daily activities. By 12 months, however, this association was no longer evident, potentially because patients had become familiar with, and confident in, their device over time.

Participation in social activities was associated with higher physical QoL at 12 months and improved mental QoL at both 6- and 12-month post-implantation. Likewise, social difficulties related to the device were associated with emotional well-being, social functioning, pain, and general health, as measured by SF-36 in a Greek sample of 150 PPM recipients [8]. Maintaining an active social life contributes to improved physical functioning over time, potentially by promoting mobility, routine, and engagement in the natural rhythm of life. Moreover, social interaction provides emotional support, reduces feelings of isolation, and fosters a sense of belonging, which positively influences mental well-being. Conversely, social isolation in cardiac patients may diminish QoL by exacerbating fatigue and hindering effective communication with healthcare providers, thereby contributing to suboptimal disease management [8,23,24]. Possibly, physical improvements associated with social participation may not be evident at 6 months post-implantation, due to ongoing recovery and limited patient adaptation, with benefits becoming more apparent over time.

Reduced anxiety about device function was associated with better physical QoL at 12 months, whereas reduced anxiety about rhythm disorders was associated with better mental QoL at both 6 and 12 months. The association between reduced anxiety about device function and improved physical QoL at 12 months may be attributed to increased physical activity levels, unimpeded by fear or functional limitations. The association between reduced anxiety about device function and improved mental QoL at both 6 and 12 months suggests that emotional reassurance concerning heart rhythm stability plays a key role in alleviating psychological distress and enhancing mental well-being. Moreover, diminished device-related anxiety may improve patient-provider interactions, treatment adherence, and ultimately, enhance patients’ QoL [7,8]. Of note, PPM implantation represents a significant bodily intervention that may disrupt psychosocial adaptation and contribute to emotional disturbances [25,26].

Both mental and physical QoL dimensions at 6 and 12 months after PPM implantation were positively associated with patients remaining physically active, and negatively associated with requiring assistance for daily activities. Patients capable of performing daily tasks are likely to experience increased confidence, autonomy, and self-efficacy, which contribute to improved mental health, while physical activity concurrently supports cardiovascular fitness and overall physical recovery. Maintaining independence enables older adults with chronic conditions to preserve their self-concept and psychological well-being, despite health challenges [27]. Equally important is the assessment of mental health in cardiac patients, as several key difficulties affect their lives, including the loss of prior relationships or roles, disease-related physical limitations, and the urgent need for risk-factor modification [28].

Last but not least, QoL is closely linked to rhythm management. More thoroughly, QoL offers an indirect, experiential measure of patients’ values and priorities by capturing their perceptions of the impact of health status and treatments on physical functioning and emotional well-being [7,8]. The United States Food and Drug Administration’s Center for Devices and Radiological Health acknowledges the importance of patient-preference data in informing decision-making [29]. Incorporating QoL into pre-implant assessments allows clinicians to understand individual patient needs, preferences, and risk profiles, thereby enabling tailored device selection. For example, leadless systems may be preferable for patients whose cosmetic concerns or early mobility limitations could adversely affect physical outcomes, including highly active individuals or those requiring rapid restoration of functional independence. Traditional transvenous systems may be preferable for patients who require frequent device upgrades or greater programmability, due to complex rhythm management [30].

Apart from device selection, assessing QoL before and after implantation equips clinicians with insights to identify patients at risk of suboptimal adaptation, personalize counseling, and optimize follow-up. Our findings highlight that modifiable factors are associated with post-implant QoL and should be incorporated into a patient-centered care approach. QoL assessment complements clinical measures by reflecting improvements in daily functioning, thus indicating real-world treatment efficacy.

Though the focus of the present study was to explore QoL and patients’ perspectives and behaviors, there is a need to acknowledge potential confounding factors, which include socioeconomic status, perceived social support, and psychological disturbances such as anxiety or depression. These variables may affect QoL and potentially obscure the true impact of PPM implantation. Although left ventricular ejection fraction and New York Heart Association (NYHA) functional classification offer valuable information on cardiac function, our research did not include these clinical parameters. Consequently, it was not possible to determine whether improvements in QoL were directly attributable to enhanced cardiac function. Also, Willy et al., in their systematic review, showed that many studies lacked or did not report data on patients’ baseline functional status (NYHA class) or left ventricular function, despite these factors being well-established determinants of QoL [20]. Future studies should, therefore, systematically incorporate baseline functional status, including NYHA class and left ventricular function, to enable a clearer understanding of how cardiac functional parameters interact with QoL outcomes in PPM populations.

Limitations of the study

Repeatedly observing the same participants over time allows for the analysis of within-individual changes in QoL. However, this design cannot definitively establish causality. Furthermore, using non-probability (convenience) sampling limits the sample’s representativeness, which, in turn, restricts the generalizability of the study findings to the broader population of PPM recipients in Greece.

The relatively small and easily accessible sample may limit the statistical power of the study, reducing the likelihood of detecting significant differences between variables. A larger sample size generally enhances the ability to identify such differences if they exist. Equally important is the recognition that self-report instruments for assessing QoL are prone to recall and response biases, which can affect study validity and introduce variability unrelated to actual health status. Given that the SF-36 was interviewer-administered, the possibility of interviewer-induced response bias cannot be excluded. The interviewer’s presence or interaction style may have influenced participants’ responses, representing a methodological limitation that should be considered when interpreting the study’s findings.

## Conclusions

Results revealed that the physical QoL of PPM recipients remained limited, whereas the mental QoL improved markedly within the first year following implantation. Both the mental and physical dimensions, at 6 and 12 months after PPM implantation, were associated with patients remaining physically active and not requiring assistance with daily activities. Reduced anxiety about device functioning was associated with better physical QoL at 12 months, while reduced anxiety about rhythm disorders was associated with better mental QoL, both at 6 and 12 months. Participation in social activities was associated with better physical QoL at 12 months and better mental QoL at both 6 and 12 months. Adherence to regular follow-up visits and to antiarrhythmic therapy was positively associated with better physical QoL at 6 and 12 months. Furthermore, in terms of physical dimension, at 6 months, those carrying the PPM ID card reported better QoL.

Assessing QoL both before and after PPM implantation allows healthcare providers to measure the intervention’s true impact on the patient’s well-being. Pre-implant assessment establishes a baseline, while post-implant evaluation reveals improvements or declines in QoL. A comprehensive understanding, by clinicians, of patients’ subjective experiences enables the formulation of realistic expectations and the personalization of pacing therapy to align with patients’ individual objectives. Incorporating the patient’s perspective into clinical decision-making enhances guidance during rehabilitation and aids in identifying those who may benefit from additional support. This study contributes valuable knowledge regarding patient experiences and advances a patient-centered framework for evaluating pacing therapy.
